# Is visceral fat thickness related to outcomes in cancer patients with pulmonary embolism?

**DOI:** 10.1590/1806-9282.20240649

**Published:** 2025-06-02

**Authors:** Filiz Taşçı, İsmail Ataş, Mümin Murat Yazıcı, Enes Güler, Özlem Bilir

**Affiliations:** 1 Recep Tayyip Erdoğan University, Training and Research Hospital, Department of Radiology – Rize, Türkiye.; 2 Rize State Hospital, Department of Emergency Medicine – Rize, Türkiye.; 3 Recep Tayyip Erdoğan University, Training and Research Hospital, Department of Emergency Medicine – Rize, Türkiye.; 4 Ministry of Health Edirne Sultan Murat 1 State Hospital, Department of Emergency Medicine – Edirne, Türkiye.

**Keywords:** Pulmonary embolism, Cancer, Visceral fat, Helical computed tomography

## Abstract

**OBJECTIVE:**

The aim of this study was to determine the relationship between visceral fat thickness measurements obtained via computed tomography and clinical outcomes in order to predict the outcomes of oncology patients diagnosed with acute pulmonary embolism at the emergency department.

**METHODS:**

This study was conducted retrospectively by examining 75 cancer patients diagnosed with acute pulmonary embolism at the emergency department between 2019 and 2022. Visceral fat thickness was evaluated using the axial sections of computed tomography pulmonary angiography taken at the level of the first lumbar vertebra at the time of diagnosis. Clinical scores, hemodynamic status, laboratory and radiological parameters, length of hospital stay, and in-hospital and 90-day mortality were assessed.

**RESULTS:**

Notably, 20% of the patients included in the study were hemodynamically unstable at the time of presentation. In-hospital mortality occurred in 25% of the patients with breast cancer, while 90-day mortality was observed in 30% of those with lung cancer. The median visceral fat thickness obtained from computed tomography pulmonary angiography was 113 cm^2^. The patients’ visceral fat thickness values statistically significantly differed according to the presence of 90-day late-period mortality (p=0.025).

**CONCLUSION:**

Muscle and fat tissue areas obtained from imaging modalities used in the diagnosis of critical conditions, such as pulmonary embolism and cancer, have emerged as significant biomarkers for mortality in the literature. In light of our findings, we consider that visceral fat thickness can predict late-period mortality in oncological patients with pulmonary embolism.

## INTRODUCTION

With the increase in cancer prevalence across the world, deep vein thrombosis (DVT) and pulmonary embolism (PE) have become important causes of mortality and morbidity^1^. The most important etiological factors are risk factors that vary depending on the patient's age, tumor type, thrombogenic potential of the tumor, and treatment methods applied for primary disease^2^.

Clinical probability scores, D-dimer, and computed tomography pulmonary angiography (CTPA) findings are the fundamental elements in determining the basic strategies to be followed in the diagnosis and treatment of PE. Cancer patients with PE have a worse prognosis due to their elevated D-dimer levels and risk of thrombosis as a result of the natural activation of the coagulation cascade^3^. In addition to being used as the gold standard imaging method for the diagnosis of PE, CTPA is also effective in the exclusion of fatal thoracic syndromes and the evaluation of right ventricular function and complications that may arise due to primary tumors in emergency cases^4^. Furthermore, in recent studies, body composition characteristics evaluated in muscle and fat tissue areas on computed tomography (CT) have been defined as new imaging markers for the prediction of the outcomes of individuals with both PE and critical diseases, such as malignancies^5^.

The variability in the distribution and quantity of fat tissue throughout the body entails certain cardiovascular and metabolic risks. In particular, visceral fat tissue is a structure effective in the release of inflammatory cytokines^6^. Therefore, the prognostic value of parameters obtained as a result of measurements made through imaging examinations is of great importance in various medical disciplines^7,8^. The current study performed visceral fat thickness (VFT) measurements in cancer patients diagnosed with PE at the emergency department in order to determine the relationship between these measurements and short- and long-term clinical outcomes.

## METHODS

The study was conducted retrospectively by examining cancer patients with acute PE detected on CTPA at the emergency department of a tertiary hospital between January 2019 and December 2022.

The local computer-based hospital information management system, where patient records are kept, was used to collect the clinical and radiological data of patients with acute PE who were included in the study. Patients younger than 18 years, those with incomplete clinical and laboratory data, those with non-diagnostic CTPA findings (movement artifacts, no contrast material injection, or errors during injection), and those who were not followed up for a 90-day period after diagnosis were excluded from the study. As a result, 75 patients who were diagnosed with acute PE at the emergency department and had a malignancy diagnosis in their medical history were included in the study.

Patients considered to have acute PE based on their current symptoms at the emergency department are classified as hemodynamically stable or unstable according to their clinical condition. Those with unstable hemodynamic parameters are scanned with CTPA for diagnostic purposes, provided that stabilization is achieved. Patients who could not be stabilized hemodynamically were excluded from the current study. The primary endpoint of the study was mortality that occurred during the follow-up and treatment of patients hospitalized with a diagnosis of PE (early-period mortality), and the secondary endpoint was mortality within 90 days after diagnosis (late-period mortality).

### Clinical parameters

pon their presentation to the emergency department, the patients’ blood pressure, heart rate, and saturation values were recorded, along with their medical history and demographic data. The Wells, revised Geneva^9^, and Simplified Pulmonary Embolism Severity Index (sPESI)^10^ scores were calculated. In addition, D-dimer (0–0.5 μg/mL), troponin-I (0–34.2 ng/L), and lactate (0.5–1.6 mmol/L) measurements were obtained. The study assessed mortality rates for all causes throughout the hospital stay and the subsequent 90-day period.

### Imaging technique

CTPA was performed using a 16-slice CT scanner (Alexion TSX-034A, Toshiba, Shimoishigami, Otawara-Shi, Toschigi-Ken, Japan). Iodine-based contrast material (Iopromide, Ultravist, 300 mg I/mL, Bayer Schering Pharma, Berlin, Germany) was administered intravenously through the peripheral venous line at a rate of 4.0 mL s^-1^. Imaging was performed automatically as a bolus, triggering 100 Hounsfield units (HU) in the pulmonary trunk. Images were obtained at 100 kVp, 125 mAs, and 1-mm slice thickness during deep inspiration.

### Radiological findings

Each CT scan taken at the emergency department is interpreted by the radiologist on duty via remote access immediately after the scan is complete. After the initial evaluation, the CTPA images of the patients included in the study were retrospectively re-evaluated by a radiologist with 20 years of cardiothoracic experience. The diagnosis of PE was established upon observation of a pulmonary arterial contrast filling defect in at least two consecutive sections. The diameter of the main pulmonary artery (mPA), area of vascular obstruction as central (within the main and/or lobar pulmonary artery) or peripheral (including segmental and/or subsegmental pulmonary arterial branches), and degree of obstruction in the central or peripheral vessels (<50 or ≥50%) were noted. On CT, the transverse axial measurement of the diameter of the mPA was performed at the level of bifurcation.

Using CTPA taken without cardiac synchronization, the right ventricular diameter was determined by measuring the distance from one end to the other end of the inner surface of the ventricular wall at the valve level during the same cardiac cycle and on the same axial image^11^.

### Visceral fat thickness measurement

**Figure 1:**
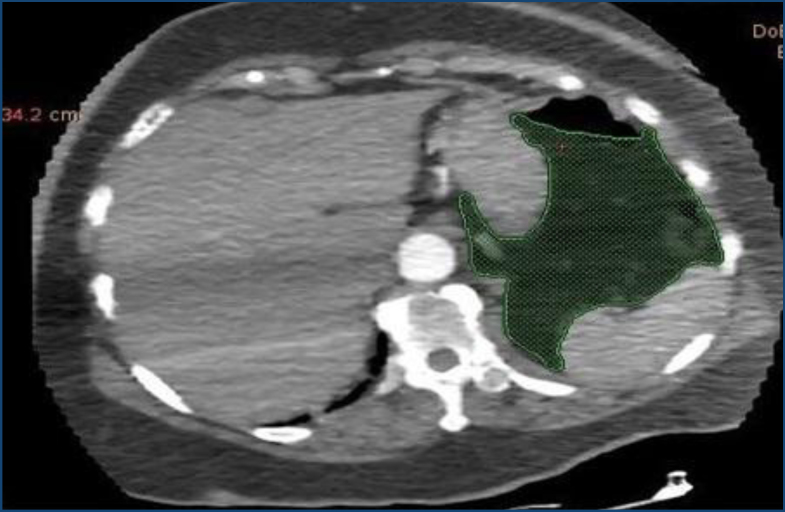
Measurement of visceral fat thickness using an axial section through the middle of the first lumbar vertebra (L1). The visceral fat thickness was evaluated at the level of the first lumbar vertebra (L1) on the axial computed tomography scans using a semi-automatic program. The green area shows the visceral fat tissue of a sample case.

VFT was measured semi-automatically using freely available GE, General Electric Company Healthcare AW4, software, USA. Area evaluation was performed as recommended in similar studies, using an axial section through the middle of the first lumbar vertebra (L1) (Figure 1)^8,12^.

### Statistical analysis

All statistical analyses were performed on Jamovi v. 1.6 software (Jamovi Project Computer Software, version 1.6. Sydney, Australia). Type 1 errors were regarded as 5% for all comparisons. The Shapiro-Wilk test was used to evaluate the normality of the data distribution. For continuous variables, non-normally distributed data were expressed as median and interquartile range and normally distributed data as mean±standard deviation (SD) and minimum–maximum values. Categorical data were expressed as frequency (n) and percentage (%) values. The comparisons of continuous variables were undertaken using the t-test for normally distributed groups and the Mann-Whitney U test for non-normally distributed groups. Categorical variables were compared utilizing the chi-square test.

## RESULTS

A total of 75 oncological patients diagnosed with acute PE were identified during the study period. The mean (±SD) age of the patients was 66.6±10.8 years, and 52% were male (Table 1). The most common malignancy was lung cancer (28%), followed by breast cancer (12%). The main indication for CTPA was the presence of complaints such as shortness of breath (76%) and chest pain (6.7%), along with high PE probability scores and elevated D-dimer values according to age (median: 3,200 μg/L). In addition, 9.3% of the patients had a previous history of PE, while 2.7% had a history of DVT. Notably, 20% of the patients were hemodynamically unstable at the time of presentation. The median sPESI score, a marker of 30-day mortality, was determined to be 2. While 21.3% of the patients had in-hospital mortality, 13.3% died within the 90-day period following their discharge from the hospital. Twenty-five percent of the patients with in-hospital mortality had breast cancer, and 30% of those who died within the 90-day period had lung cancer.

**Table 1:** Patients’ demographic data, computed tomography findings and measurements, and baseline characteristics.

Characteristics, n=75		Value
Gender		
	Male, n (%)	39 (52.0%)
	Female, n (%)	36 (48.0%)
	Age (years), mean±SD (min–max)	66.6±10.8 (38–90)
Comorbidities		
	Hypertension, n (%)	36 (48.0%)
	Diabetes, n (%)	11 (14.7%)
	CAD, n (%)	32 (42.7%)
	CHF, n (%)	2 (2.7%)
	COPD, n (%)	10 (13.3%)
	Atrial fibrillation, n (%)	18 (24.0%)
	Stroke, n (%)	11 (14.7%)
	Dementia, n (%)	5 (6.7%)
	History of PE, n (%)	7 (9.3%)
	History of DVT, n (%)	2 (2.7%)
Vital signs		
	SBP (mmHg), median (IQR)	120 (110–130)
	DBP (mmHg), median (IQR)	80 (70–80)
	Pulse (min), median (IQR)	90 (80.5–103)
	OS (%), median (IQR)	90 (88–95)
Laboratory parameters		
	Troponin I (ng/L), median (IQR)	17.4 (0.1–40.3)
	D-dimer (μg/L), median (IQR)	3,200 (1,700–5,700)
	Lactate (mmol/L), median (IQR)	2.1 (1.6–2.8)
	Wells score, median (IQR)	2 (1–3)
	sPESI, median (IQR)	2 (1–3)
	Geneva score, median (IQR)	3 (3–4)
	YEARS score, median (IQR)	1 (0–1)
Mortality		
	Early period*, n (%)	16 (21.3 %)
	Late period&, n (%)	26 (34.7 %)
CT findings		
	Pleural fluid, n (%)	28 (37.3%)
	Pericardial fluid, n (%)	11 (14.7%)
	Atelectasis, n (%)	27 (36.0%)
	Hampton's hump, n (%)	27 (36.0%)
CT measurements		
	MPA diameter (mm), mean±SD (min–max)	28.4±3.8 (20.3–40.0)
	RV diameter (mm), mean±SD (min–max)	40.9±6.4 (17.0–56.3)
	VFT (mm), median (IQR)	113 (73.9–174)
Pulmonary embolism side		
	Right, n (%)	19 (25.3%)
	Left, n (%)	14 (18.7%)
	Bilateral, n (%)	42 (56.0%)
Occlusion percentage		
	<50%, n (%)	53 (70.7%)
	≥50%, n (%)	22 (29.3%)

SD: standard deviation; IQR: interquartile range (25p, 75p); CAD: coronary artery disease; CHF: congestive heart failure; COPD: chronic obstructive pulmonary disease; PE: pulmonary embolism; DVT: deep vein thrombus; SBP: systolic blood pressure; DBP: diastolic blood pressure; OS: oxygen saturation; sPESI: Simplified Pulmonary Embolism Severity Index; CT: computed tomography; MPA: main pulmonary artery; RV: right ventricle; VFT: visceral fat thickness.

* Early-period mortality,

& Late-period mortality.

In the images re-evaluated by a specialist radiologist, the localization of PE was found to be central in 30 (40%) patients, segmental in 18 (24%), and subsegmental in 27 (36%). According to the CTPA findings, 36% of the patients had Hampton's hump and 29.3% had ≥50% occlusion. The mean mPA diameter was 28.4±3.8 mm, the mean right ventricular diameter was 40.9±6.4 mm, and the median VFT value was 113 cm^2^ (Table 1).

Upon evaluation of the findings obtained from CTPA taken at the time of presentation to the emergency department according to mortality status, it was determined that the VFT values were statistically significant in predicting 90-day mortality (p=0.025). Other imaging findings were not found to be statistically significant for this purpose (Table 2).

**Table 2:** Evaluation of computed tomography measurements according to mortality status.

Variables		Mortality status			p-value*	p-value&
CT findings		Survival (n=49)	Early-period mortality* (n=16)	Late-period mortality & (n=26)		
Pleural fluid, n		19	7	9	0.550	0.723
Pericardial fluid, n		6	4	5	0.188	0.416
Atelectasis, n		14	8	13	0.188	0.066
Hampton's hump, n		17	7	10	0.467	0.746
CT measurements						
	MPA diameter (mm), mean±SD (min–max)	27.9±3.2 (21.4–35.2)	29.2±4.7 (22.8–40.0)	29.3±4.7 (20.3–40.0)	0.328	0.137
	RV diameter (mm), mean±SD (min–max)	40.5±6.4 (17–53.6)	43.4±6.6 (34.0–56.3)	41.7±6.6 (30.0–56.3)	0.081	0.450
	VFT (mm), median (IQR)	130 (90–190)	102 (54.5–129)	85.8 (56.9–132)	0.058	**0.025**
Pulmonary embolism side						
	Right, n	15	3	4		
	Left, n	6	5	8	0.332	0.095
	Bilateral, n	28	8	14		
Occlusion percentage						
	<50%, n	35	11	18	0.849	0.842
	≥50%, n	14	5	8		

CT: computed tomography; SD: standard deviation; IQR: interquartile range (25th–75th percentile); MPA: main pulmonary artery; RV: right ventricle; VFT: visceral fat thickness,

* Early-period mortality,

& Late-period mortality. Statistically significant value is denoted in bold.

## DISCUSSION

Thoracic imaging allows for the detection and staging of thromboembolic complications, such as PE. In recent studies, body composition characteristics evaluated in muscle and fat tissue areas on CT have been defined as new imaging biomarkers for the prediction of the outcomes of individuals with both PE and critical diseases, such as malignancies^8^. In this retrospective study, the VFT of oncological patients with acute PE was statistically significantly greater among those with late-term mortality than in the remaining groups.

In the literature, it has been suggested that an increased amount of abdominal visceral fat plays a role in hemostasis and fibrinolysis disorders^13^. In addition, visceral fat tissue increases the risk of atherothrombotic disease and venous thromboembolism by contributing to the production of plasminogen activator inhibitor type 1 and proinflammatory cytokines^14^. However, in the current study, we determined that the VFT of patients with late-period mortality was lower than that of survivors and patients with early-period mortality. This may be related to gene expression that enables the restructuring of fat tissue in oncological patients^15^ and the increase in free fatty acids in the circulation along with the altered fat tissue catabolism^16^.

Although the risk of venous thromboembolism varies depending on the type of malignancy, it is most common in lung and breast cancers. Similarly, in this study, the most common malignancies were lung and breast cancers. Furthermore, in-hospital mortality was more prominent in patients with breast cancer, while 90-day mortality was more prominent in those with lung cancer.

Patients’ complaints at the time of presentation also consist of non-specific symptoms such as shortness of breath and chest pain that may occur due to the primary tumor. Therefore, in the evaluation of patients, it is important to use clinical scoring systems before PE testing. Despite the frequent use of the Wells and Geneva scores, there is ongoing research for the development of novel risk prediction models^17,18^. The Wells, Geneva, and YEARS scores calculated in this study indicated high probability rates for PE among oncological patients. In addition, the sPESI score, which evaluates 30-day mortality, was determined to be ≥1 in this patient group. While the in-hospital mortality rate (21.3%) was consistent with the literature, the late-period mortality rate was found to be higher at 34.7%.

Right ventricular function plays an important role in prognostic classification and determining treatment strategies in acute PE. In this respect, CT provides an alternative to echocardiography by allowing the imaging and measurement of cardiac chambers. However, rather than using CT alone, the combined use of both imaging and laboratory markers is a more effective method in the evaluation of mortality and morbidity. In the current study, the diameters of the right ventricle and mPA, the presence of Hampton's hump, the presence of pericardial and/or pleural fluid, and atelectasis were not found to be associated with mortality in the CTPA evaluations of the patients. Especially the presence of central occlusion and the degree of occlusion are the main prognostic factors in determining right ventricular function^19^. However, in our study, segmental and subsegmental occlusion was found in 60% of the patients, with the degree of occlusion being <50% in 70.3% of the patients, and the troponin values were within the reference range. These findings indicate that the right ventricular function of these patients was not affected.

## LIMITATIONS

The important limitations of the study include the single-center design, the limited number of patients, and the study population consisting of patients with different types of malignancies. In addition, although VFT depends on population characteristics, such as gender, race, and age, this distinction was not made within the scope of this study.

## CONCLUSION

In light of our findings, we consider that VFT can predict late-period mortality in oncological patients. Due to the changing fat tissue catabolism in oncology patients, we determined that patients with late-period mortality had a lower VFT value than those with early-period mortality and survivors.
